# Bladder Cancer and Cancer Stem Cells: Basic Science and Implications for Therapy

**DOI:** 10.1100/tsw.2011.117

**Published:** 2011-06-09

**Authors:** Richard T. Bryan

**Affiliations:** The School of Cancer Sciences and The Department of Public Health, Epidemiology and Biostatistics, University of Birmingham, Birmingham, UK

**Keywords:** bladder cancer, stem cells, cancer stem cells

## Abstract

Bladder cancer is the fifth most common cancer in Western society, with the global burden predicted to increase significantly in the foreseeable future. Over 90% of these bladder cancers are transitional cell carcinomas of urothelial origin (urothelial carcinomas or UCs) and at presentation, over 70% will be non–muscle-invasive or stage Ta/T1 tumours, with the remainder being muscle-invasive or stages T2-4. Bladder UC is a highly heterogeneous disease: for the 50–55% of bladder cancer patients presenting with Ta tumours, recurrence is the main issue, but for the 20–25% of patients presenting with T1 tumours, progression is the main issue. Progression to, or presentation with, muscle-invasive disease represents the critical step for patients, necessitating more aggressive therapies and carrying significantly worse survival rates. We therefore urgently require detailed molecular insights into the pathogenesis of muscle-invasive bladder cancer so that the disease can be more adequately and appropriately treated at presentation, so that progression from stages Ta/T1 can be abrogated, and so that the risk of recurrence following treatment can be minimised. The recently identified bladder cancer stem cells are considered to be mediators of resistance to current therapies and therefore represent strong candidate biological targets. The aim of this review is to discuss the background and basic science of such cells, and the implications for current and future therapies.

## INTRODUCTION

Bladder cancer is the fifth most common cancer in Western society, with a global incidence of over 356,000 new cases per year and a prevalence estimated at 2.7milion cases[1,2]. The global burden of bladder cancer is predicted to increase significantly in the foreseeable future as a result of population aging and the increasing world population, together with the progression of the tobacco epidemic and increasing exposure to occupational carcinogens in developing countries[2]. In the U.K., we observe 10,200 new cases and 5,000 deaths per year[3]. Over 90% of these bladder cancers are transitional cell carcinomas (TCC) of urothelial origin (urothelial carcinomas or UCs) and at presentation, over 70% will be non–muscle-invasive or stage Ta/T1 tumours, with the remainder being muscle-invasive or stages T2-4[1,4,5,6].

Bladder UC is a highly heterogeneous disease[7]: for the 50–55% of patients presenting with Ta tumours, recurrence is the main issue, occurring in up to 80% of cases[1,4], whereas for the 20–25% of patients presenting with T1 tumours, progression is the main concern, occurring in 45% of cases[1,4]. However, it is progression to, or presentation with, muscle-invasive disease that represents the critical step in the disease course, necessitating more aggressive therapies (including radical surgery, chemotherapy, or radiotherapy), and carrying a 5-year survival rate of only 27–50% (compared with over 73% for Ta/T1 disease) and a median survival of only 7–20-months for patients with unresectable metastatic disease[4,8,9]. However, despite treatment success in many patients, these aggressive therapies still represent crude approaches that carry significant morbidity and mortality themselves[6,10,11,12,13,14].

We therefore require more detailed biological and molecular insights into the pathogenesis of muscle-invasive bladder cancer so that the disease can be more adequately and appropriately treated at presentation, so that progression from stages Ta/T1 can be abrogated, and so that the risk of recurrence and death following treatment can be minimised. To achieve this, we must consider the genetic and epigenetic alterations involved in tumour development, and the molecular pathways associated with these alterations, which subsequently drive these tumours towards epithelial-to-mesenchymal transition (EMT) and ultimately towards a "cancer stem cell" phenotype[15,16]. In this regard, stem cell and cancer stem cell pathways therefore represent important candidate biological targets[13,17,18,19]: the relative insensitivity of such cells to therapy[20] (even the latest "targeted" therapies) and their considered ability to metastasize highlight the need for us to attack their specific vulnerabilities in order to deliver durably effective cancer treatments[7,21]. The former areas of research have been reviewed in detail elsewhere by significant workers in the field and our understanding has developed greatly[9,16,20,21,22,23,24,25], and several reviews cover the general cancer stem cell topic in detail[13,19,20,26,27]. Here, I shall attempt to discuss the key cancer stem cell concepts, background, and basic science that are of relevance for bladder UC, and the implications for current and future therapies.

## STEM CELLS AND CANCER STEM CELLS

Whilst solid tumours can be reduced in size or totally removed, disease progression or relapse often occurs[13,19]. These phenomena may be explained by the persistence of residual tumour-initiating cells (TICs) and tumour-maintaining cells, and such cells have been reported in a variety of human malignancies (breast, brain, prostate, lung, pancreas, etc.) since their original identification in leukaemia[13,28,29]. It is proposed that these cells are the "original cells" of a tumour, responsible for tumorigenesis, tumour differentiation, tumour maintenance, tumour spread, and tumour relapse[13,17,19,27,30]. These properties are analogous to a stem cell, the original cell of an organ and responsible for organogenesis and organ maintenance, and so these cells have been termed "cancer stem cells" (CSCs)[13,17,19,27]. CSCs are thus a subset of tumour cells that have the ability to self-renew and to generate all of the heterogeneous cells that comprise a tumour[21,29]. It has been suggested that it is the CSCs that need to be eradicated to provide long-term disease-free survival; yet, as mentioned above, it appears that CSCs are more resistant to current therapies[18,21,27,31].

Self-renewal and differentiation capacity are the hallmark traits of stem cells, mirrored by the high proliferative capacity and phenotypic plasticity of tumour cells[13,17]. These parallel properties have led to the hypotheses that:

1. Cancer cell populations may include CSCs: rare cells, with indefinite potential for self-renewal, and driving tumorigenesis[17,19].
2. Tumours may originate from the transformation of normal stem cells, since stem cells have a life span long enough to accumulate the number of mutations that cause malignant transformation[13,19,31,32].
3. Similar signalling pathways may regulate stem cells and cancer cells[13,17,19].

The origin of CSCs has not been established, especially at which level of cell lineage a cell may be turned into a CSC by one or more mutations[13]. These origins are likely to vary from one tumour type to another[20]. However, I and others propose that the development of CSCs in bladder UC most likely occurs following EMT[16,33] as has been demonstrated in other epithelial malignancies[15]. In the laboratory, the occurrence of CSCs varies according to tumour type and to the specific experimental systems used, and controversy continues regarding the frequency and even the existence of CSCs[18,29]. However, the evidence does favour the existence of subpopulations of cells in some solid tumours that satisfy the functional criteria of CSCs[26,29]. It has also been suggested that a dynamic equilibrium may exist between CSCs and non-CSCs within tumours, with non-CSCs from different tumours possessing differing susceptibilities to becoming CSCs[29]; optimal therapeutic regimes would therefore need to incorporate agents to target both CSCs and non-CSCs[29].

A better understanding of benign bladder stem cell biology may enable us to identify and characterise bladder CSCs[13,19], but, to date, definitive cell surface immunophenotypes have not been defined for most stem cells. A study by Signoretti et al. suggested the notion that umbrella and basal/intermediate cells were either entirely independent lineages capable of self-renewal or were maintained by a common p63-negative progenitor cell[34]. Therefore, until recently, bladder stem cells had not been clearly identified[13,27,32,35]. However, it was believed that there was a proliferative basal cell layer that contained a pool of stem cells responsible for replenishing the more differentiated umbrella cells that form the superficial layer of the normal urothelium[21,36], and this hypothesis was supported by the existence of monoclonal patches covering the bladder[36]. Thangappan and Kurzrock investigated this in more detail and demonstrated the existence of three distinct cell types in the normal urothelium: basal cells with stem cell qualities; an intermediate population of committed progenitors with limited proliferative capacity responsible for amplification of each stem-cell division and akin to the “transit amplifying” cells of the skin, residing in the basal and intermediate compartments of the urothelium; and the terminally differentiated superficial umbrella cells[36].

## BLADDER STEM CELLS AND CANCER STEM CELLS

As discussed above, stem cells are able to reproduce themselves, have a high capacity for cell division and a long life, and can produce at least one type of highly differentiated descendent[32,35]. Stem cells are also slower-cycling cells that retain nucleic acid labels for longer periods of time, in contrast to more rapidly cycling cells that incorporate such labels faster, mature, and then die[32]. Thus, they have also been termed “label-retaining cells” or LRCs[32,37]. The urothelial stem cell “niche” is the stem cell’s anatomical and functional microenvironment, protecting it from differentiation, loss of self-renewal capacity, and apoptosis[13] and, prior to the studies of Thangappan and Kurzrock[36], this had already been presumed to be along the basement membrane[18,32,38]. LRCs had been localised to this basal layer, with these cells being associated with poorly differentiated UCs and demonstrating stem cell-like properties[7,32]. These cells also shared cytokeratin and cell surface marker expression profiles with bladder TICs[18]. Furthermore, cells derived from this layer were highly tumorigenic cells (HTCs) with enhanced growth potential and multipotency[38]. Such cells also show regulation of Wnt pathway components in a pattern expected to stimulate Wnt signalling activity, as suggested by up-regulation of some Wnt targets (e.g., *myc*) and accumulation of the Wnt pathway effector, β-catenin[38]. The Wnt signalling pathway regulates stem cell maintenance and differentiation in a variety of tissues[13,19,39], and it was therefore proposed that during tumorigenesis, some UCs have a basal population at the tumour-stroma interface that resemble benign urothelial stem cells and that may represent CSCs[38]. This population differentiates as it moves away from this basal compartment and loses tumour-forming potential upon differentiation[38]. Stem-like properties may be subsequently reproduced at the invasive front, possibly within another niche at this tumour-stroma interface[13,38].

For some time, however, CSCs had not been clearly identified in bladder UC[13,27,32,35], although their possible existence was feasible since the disease is characterised by frequent recurrences and a high risk of progression for some groups of patients[31]. Some high-grade UCs appeared to possess the molecular imprints of stemness, demonstrating a TIC gene expression signature[18] or activity in gene sets that was associated with human embryonic stem cell identity[17,38], or nuclear accumulation of β-catenin (evidence of Wnt signalling)[18]. Evidence in support of the existence of CSCs in bladder UC could also be found elsewhere in the literature: Oct-3/4 is a transcription factor that is a key regulator of pluripotency and self-renewal in embryonic stem cells and is also expressed in bladder UCs, with intense expression associated with disease progression and metastasis[40]. Some workers also claimed to have isolated CSCs from bladder tumours and bladder cancer cell lines[41,42], and bladder cancer cell populations expressing the putative stem cell marker CD44[27] demonstrate an enhanced ability to form tumours in mice[18].

More recently, other workers also identified the existence of cancer cells showing stem cell features in human bladder UCs[43]. Moreover, different populations of CSCs may reside in the same tumour with diverse tumorigenic potential and distinct genetic anomalies[43]. Therefore, there now appears to be compelling evidence to support the existence of CSCs in bladder UCs, a notion that potentially explains tumour resistance to chemo- and radiotherapy, and disease relapse. Workers are now trying to establish a molecular profile for bladder CSCs to enable their identification within tumour specimens. Preliminary reports appear to suggest that this profile mirrors that seen in the basal and intermediate cells of the normal urothelium; CD44, CD47, and cytokeratins 5 and 17 appear to have a role[7,16,21]. Chan et al. have subsequently identified a gene signature associated with CD44-positive CSCs and also consider CD47 to be a promising target[7]. Our own work and that of others is investigating, in addition, another molecule expressed by basal cells of the normal urothelium and that is also expressed by more aggressive bladder UCs: P-cadherin[33,44,45]. However, it remains unclear whether these various different molecular profiles are inherited from a stem cell-of-origin, or are reactivated during tumour progression[17].

## DISCUSSION AND IMPLICATIONS FOR THERAPY

As discussed above, evidence now seems to support the CSC paradigm for bladder cancer. However, one can also speculate that these events could occur via other molecular mechanisms and pathways, with CSCs being responsible for a minority[13,46], and I thus propose the stepwise process illustrated in Fig. 1. Instead, some workers suggest that tumour heterogeneity arises from genetic and epigenetic differences between tumour cells, resulting from selective pressure during tumorigenesis[18], although the genetic instability operating during the later stages of tumour progression may be so rampant as to outpace this selection with distinct subpopulations generated more rapidly than they can be eliminated[20]. It has also been suggested that UCs arise from more differentiated cells, and self-renewal capacity may be acquired secondarily by inactivation of *p53* and *RB1* function[13,46]. Equally, the surrounding microenvironment of a UC may play a very significant role[21], possibly even inducing a transitory or reversible CSC-like state[26]: EMT may drive the development of CSCs[20], yet EMT itself is reversible with mesenchymal-to-epithelial transition (MET) potentially favouring a cell's colonisation of a distant site, a site that no longer "emits" the EMT-inducing signals that the cell benefited from in the primary tumour[20]. Whether the CSC state reverses in a similar setting and fashion remains unknown, but such tumour-stroma interactions highlight the importance of the tumour microenvironment for all cancer cells, not just CSCs[20]. Moreover, manipulating the tumour microenvironment may represent an important route to controlling EMT and CSCs[20,21].

The CSC paradigm also needs to be reconciled against the existing proposed models of urothelial carcinogenesis. The traditional "field change", "field cancerization", or "oligoclonal" theory assumes multiple changes in the urothelium resulting from urinary carcinogens, with many transformed cells evolving independently into genetically unrelated tumours in a synchronous, metachronous, and multifocal fashion[47]. However, the identification of the specific multiple and complex chromosomal alterations in bladder TCC has led to the development of the "clonal" theory of bladder cancer pathogenesis,

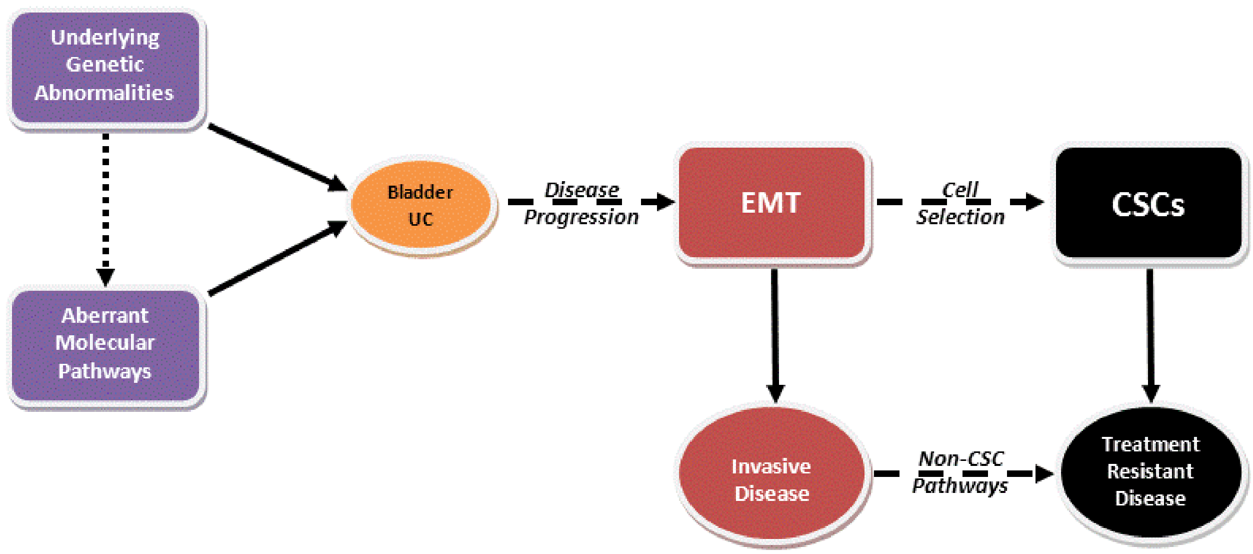

**FIGURE 1.** A proposed model for the development of bladder UC CSCs.

where it is postulated that multifocal and recurrent tumours evolve from a single transformed cell from which all progeny share several identical genetic mutations[47,48]: a number of early "hits" with subsequent losses of "forerunner genes" results in the selection and vast clonal expansion (by intraepithelial spreading) of mutant cells with a growth advantage; following further "hits", these cells become dysplastic, and may halt their differentiation and acquire a capacity for regenerating tumour growth[27,31,32,49,50]. If we now return to the original three hypotheses:

1. "Cancer cell populations may include CSCs". Bladder cancer cell populations do appear to include CSCs, although their abundance remains unknown.
2. "Tumours may originate from the transformation of normal stem cells[13,19,31,32]". This may be the case in other malignancies, but appears less likely for bladder UCs. As discussed above, some bladder tumours do demonstrate Wnt signalling activity[38], but the majority do not[44,51], and stem-like characteristics are not seen until late in the disease. Clonality appears to dominate in the early stages, with progression resulting in genetic instability, heterogeneity, and EMT. It appears that CSCs may evolve at this stage, and possibly even multiple CSC subpopulations in a model similar to field cancerization with tumour microenvironments across the tumour mass each generating their own distinct CSCs.
3. "Similar signalling pathways may regulate stem cells and cancer cells[13,17,19]". As discussed above, this appears less likely for bladder UC.

From a treatment aspect, it is sensible to consider that the elimination of CSCs will yield more durable therapeutic responses in bladder UC[7,21]. In addition, CSCs may be responsible for tumour dormancy whereby latent tumour cells persist after seemingly successful treatment, only to recur years or decades later and result in life-threatening disease[20]. The next steps for research lie in validating a group of markers that identify bladder CSCs so that their abundance in this setting can be clarified and so that the clinical problem can be more clearly defined. We may then have to consider identifying therapies that are able to specifically target CSCs, since these cells appear to be equipped with a variety of intrinsic properties that enable them to survive treatment with current therapies[21]. Unfortunately, the answers are unlikely to lie with the current so-called "targeted therapies", such as cetuximab, since an epithelial phenotype is required for response by bladder UCs *in vitro*[52], and loss of the epithelial cell adhesion molecule and tumour suppressor E-cadherin correlates with a poor response[53,54]. Tumour-stroma interactions and the tumour microenvironment appear to be of increasing importance in this setting, and identifying the events that accompany MET could be highly significant and lead to specific anti-CSC therapies. Tailored approaches may therefore be required on a patient-by-patient basis to identify the existence of CSCs within a particular tumour, to subsequently identify the different CSC populations, and then to utilise therapies to eradicate CSCs along with non-CSCs within that tumour. Effective and durable treatment may thus have to rely on increasingly complex combinations of different classes of agents. As well as the discovery of new therapeutics, the utilisation of new platforms (e.g., next-generation sequencing) to further characterise tumours alongside existing conventional histopathology may also be required.

## CONCLUSIONS

It is highly relevant to the growing worldwide bladder cancer patient population that we and others continue to investigate the existence, abundance, and molecular pathogenesis of bladder cancer stem cells. In the future, our therapeutic approaches should target the underlying genetic abnormalities seen in bladder cancer, the subsequent aberrant molecular pathways, and the evolution of CSCs to further optimise the management of this significant disease.
